# Effects of Fucoidans from Five Different Brown Algae on Oxidative Stress and VEGF Interference in Ocular Cells

**DOI:** 10.3390/md17050258

**Published:** 2019-04-30

**Authors:** Philipp Dörschmann, Kaya Saskia Bittkau, Sandesh Neupane, Johann Roider, Susanne Alban, Alexa Klettner

**Affiliations:** 1 Department of Ophthalmology, University Medical Center, University of Kiel, Arnold-Heller-Str. 3, Haus 25, 24105 Kiel, Germany; Philipp.Doerschmann@uksh.de (P.D.); johann.roider@uksh.de (J.R.); 2 Department of Pharmaceutical Biology, Pharmaceutical Institute, University of Kiel, Gutenbergstraße 76, 24118 Kiel, Germany; kbittkau@pharmazie.uni-kiel.de (K.S.B.); sneupane@pharmazie.uni-kiel.de (S.N.); salban@pharmazie.uni-kiel.de (S.A.)

**Keywords:** fucoidan, age-related macular degeneration, VEGF, oxidative stress, *Saccharina latissima*, *Fucus vesiculosus*, *Fucus distichus* subsp. *evanescens*, *Fucus serratus*, *Laminaria digitata*

## Abstract

Background: Fucoidans are interesting for potential usage in ophthalmology, and especially age-related macular degeneration. However, fucoidans from different species may vary in their effects. Here, we compare fucoidans from five algal species in terms of oxidative stress protection and vascular endothelial growth factor (VEGF) interference in ocular cells. Methods: Brown algae (*Fucus vesiculosus, Fucus distichus* subsp. *evanescens, Fucus serratus, Laminaria digitata, Saccharina latissima*) were harvested and fucoidans isolated by hot-water extraction. Fucoidans were tested in several concentrations (1, 10, 50, and 100 µg/mL). Effects were measured on a uveal melanoma cell line (OMM-1) (oxidative stress), retinal pigment epithelium (RPE) cell line ARPE19 (oxidative stress and VEGF), and primary RPE cells (VEGF). Oxidative stress was induced by H_2_O_2_ or tert-Butyl hydroperoxide (TBHP). Cell viability was investigated with methyl thiazolyl tetrazolium (MTT or MTS) assay, and VEGF secretion with ELISA. Affinity to VEGF was determined by a competitive binding assay. Results: All fucoidans protected OMM-1 from oxidative stress. However, in ARPE19, only fucoidan from *Saccharina latissima* was protective. The affinity to VEGF of all fucoidans was stronger than that of heparin, and all reduced VEGF secretion in ARPE19. In primary RPE, only the fucoidan from *Saccharina latissima* was effective. Conclusion: Among the fucoidans from five different species, *Saccharina latissima* displayed the most promising results concerning oxidative stress protection and reduction of VEGF secretion.

## 1. Introduction

Fucoidans are sulfated polysaccharides found in the cell walls of brown algae. Common to all fucoidans is a high amount of L-fucose, yet their structures are complex and variable among different species [1]. Fucoidans have been described as exerting interesting pharmacological activities including, e.g., anti-inflammatory, antitumorigenic, and anti-angiogenic effects [1,2]. In particular, a fucoidan has been found to be potentially beneficial in age-related macular degeneration (AMD), the most common cause of blindness and severe vision loss in the Western world [3,4]. AMD is a disease of the elderly in which photoreceptors and retinal pigment epithelial cells of the macula, the area of high acuity vision, degenerate, and, in the more severe exudative subtype of the disease (wet AMD), vessels grow from the choroid under and into the retina. These immature vessels are leaky and lead to fluid accumulation and tissue destruction. The pathogenesis of AMD is complex and not fully elucidated. It is a multifactorial disease and several factors are involved in its pathogenesis. The most important ones are oxidative stress and, in wet AMD, the secretion of vascular endothelial growth factor (VEGF) by cells of the retinal pigment epithelium (RPE) [5,6,7]. Other factors such as impaired complement regulation, lipid dysregulation, and inflammation are also of importance for AMD development [8,9,10]. Currently, there is no cure for AMD, and the only treatment options are VEGF inhibitors, which need to be regularly injected in the eye [11]. Although these inhibitors have been a great progress in AMD therapy, long-term treatment usually cannot keep up the initial beneficial effects, and may lead to macular atrophy [12,13]. New treatment options would, therefore, be of great benefit.

Fucoidan could be of interest for the development of new AMD therapeutics, since it has been described to be anti-inflammatory, blood lipid-reducing and, most importantly, protective against oxidative stress and VEGF-inhibiting [4]. 

Our group has previously shown that commercially available fucoidan (from *Fucus vesiculosus*) exhibits interesting effects on RPE cells including reduction of VEGF secretion and reduction of angiogenesis [3]. However, the commercially available fucoidan is poorly defined, with pronounced variability in structural composition and degree of purity between batches [14,15]. Furthermore, fucoidans from different species differ in their composition and may thus exert different biological effects. This renders the search for the most suitable fucoidan for specific applications such as AMD an important quest [4,16]. 

In the current study, we compared the fucoidans of five species of brown algae (*Saccharina latissima* (SL), *Laminaria digitata* (LD), *Fucus serratus* (FS)*, Fucus vesiculosus* (FV), and *Fucus distichus* subsp. *evanescens* (FE) in terms of two important factors for AMD development, i.e., oxidative stress and VEGF secretion in ocular cells, as well as their binding affinity to VEGF. For this comparison, the algal material of all five species were harvested in summer, identically prepared, and then extracted according to the same standardized protocol, leading to the fucoidans SL, LD, FS, FV, and FE.

## 2. Results

### 2.1. Oxidative Stress Protection

#### 2.1.1. OMM-1 Cells

The potency of oxidative stress protection of the fucoidan from five different algae species was compared in two different systems. We have previously shown that commercial fucoidan from *Fucus vesiculosus* protected several uveal melanoma cells, including OMM-1, from oxidative stress induced by H_2_O_2_ [17]. In this study, we used the uveal melanoma cell line OMM-1. 

**Figure 1:**
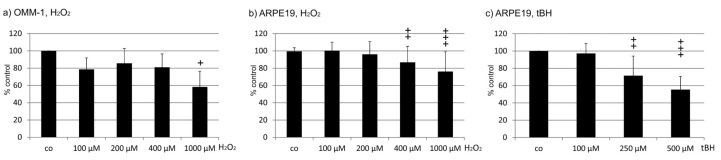
Characterization of the susceptibility of cell lines to oxidative stress. Cell viability was tested in OMM-1 (**a**) and ARPE19 (**b**) exposed to H_2_O_2_ (**a**,**b**) and tert-Butyl hydroperoxide (TBHP) (**c**). Significance was evaluated with Friedman’s ANOVA and Student’s *t*-test, + *p* < 0.05, ++ *p* < 0.01, +++ *p* < 0.001 compared to control (*n* > 3).

Prior to the experiments with fucoidans, the concentration of H_2_O_2_ causing about 50% cell death had to be evaluated. While the concentrations of 100 µM (78.67 ± 13.22%), 200 µM (85.67 ± 17.02%) and 400 µM (81.00 ± 15.51%) showed no effect on cell survival, 1000 µM displayed a significant reduction of cell viability compared to the control (1000 µM 58.33 ± 17.98%, *p* < 0.05) (Figure 1a). A concentration of 1000 µM H_2_O_2_ was therefore chosen for the following experiments. 

**Figure 2:**
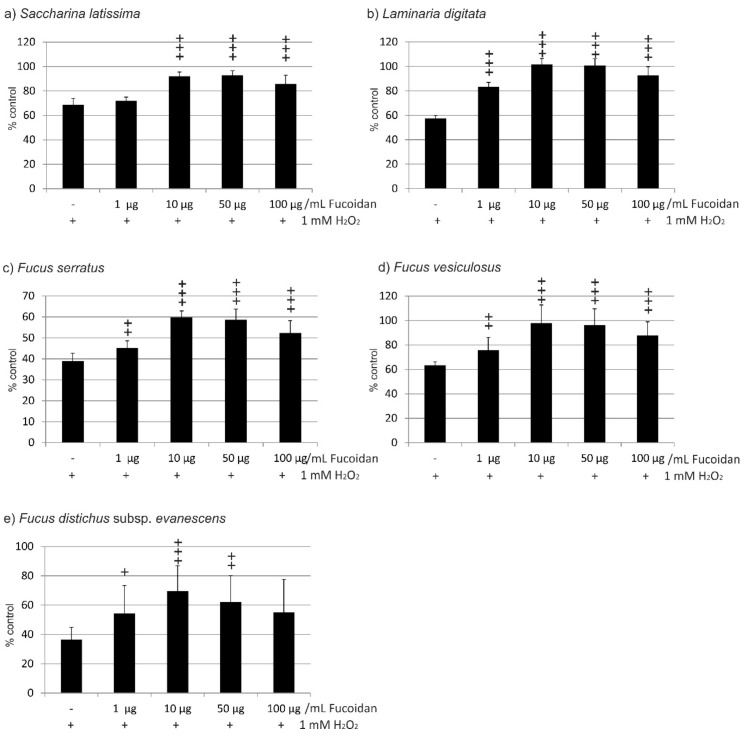
Cell viability of OMM-1 cells challenged with 1 mM H_2_O_2_ after incubation with fucoidan from (**a**) *Saccharina latissima* (SL), (**b**) *Laminaria digitata* (LD), (**c**) *Fucus serratus* (FS), (**d**) *Fucus vesiculosus* (FV), (**e**) *Fucus distichus* subsp. *evanescens* (FE). Cell viability was measured by MTS assay and is depicted as mean and standard deviation, with the control set as 100%. All fucoidans tested displayed protective effects, with the efficacy of LD > FV > SL > FE > FS. Significance was evaluated with Friedman’s ANOVA and subsequent Student’s *t*-test, + *p* < 0.05, ++ *p* < 0.01, +++ *p* < 0.001, all versus 1 mM H_2_O_2_ (*n* = 8).

In the experiments concerning the fucoidan from *Saccharina latissima*, incubation of OMM-1 treated with 1 mM H_2_O_2_ and SL fucoidan induced significant changes according to ANOVA testing. Incubation with 1 mM H_2_O_2_ resulted in a reduction of cell viability to 68.75% (±5.07). Incubation with 1 µg/mL induced no significant protection (72.00 ± 3.04%), while 10 µg/mL, 50 µg/mL, as well as 100 µg/mL all significantly increased cell viability (92.13 ± 3.41%; 93.00 ± 3.57%, and 85.88 ± 7.03%, respectively; all *p* < 0.001) (Figure 2a). In the experiments testing fucoidan from *Laminaria digitata*, incubation with 1 mM H_2_O_2_ reduced cell viability to 57.50% (±2.29). The differences between the groups treated with LD fucoidan were significant in ANOVA testing. Incubations with any concentration of LD fucoidan tested resulted in a highly significant protection of cell viability (all *p* < 0.001) (1 µg/mL 83.25 ± 3.60%; 10 µg/mL 101.75 ± 4.71%; 50 µg/mL 100.88 ± 5.51%; 100 µg/mL 92.75 ± 7.03%) (Figure 2b). Testing fucoidan from *Fucus serratus*, incubation with 1 mM H_2_O_2_ resulted in a reduction of cell viability to 39.00% (±3.67). In ANOVA testing, significant differences between the groups could be detected. FS fucoidan increased cell viability significantly, but viability remained considerably low (1 µg/mL 45.25 ± 3.35%, *p* < 0.01; 10 µg/mL 59.88 ± 3.02%, *p* < 0.001; 50 µg/mL 58.63 ± 5.10%, *p* < 0.001; 100 µg/mL 52.38 ± 5.87% *p* < 0.001) (Figure 2c). When testing the fucoidan from *Fucus vesiculosus*, incubation with 1 mM H_2_O_2_ resulted in a reduction of cell viability to 63.50% (±2.60). In ANOVA testing, significant differences between the groups could be detected. All concentrations of FV fucoidan resulted in a significantly increased viability of cells (1 µg/mL 75.75 ± 10.50%, *p* < 0.01; 10 µg/mL 97.88 ± 14.93%, *p* < 0.001; 50 µg/mL 96.36 ± 13.30%, *p* < 0.001; 100 µg/mL 87.88 ± 11.13%, *p* < 0.001) (Figure 2d). Finally, when testing the fucoidan from *Fucus distichus* subsp. *evanescens*, incubation with 1 mM H_2_O_2_ resulted in a reduction of cell viability to 36.50% (±8.44). In ANOVA testing, significant differences between the groups could be detected. FE fucoidan significantly increased viability when used at 1–50 µg/mL (1 µg/mL 54.38 ± 18.00%, *p* < 0.05: 10 µg/mL 69.5 ± 17.43%, *p* < 0.001; 50 µg/mL 62.00 ± 18.10%, *p* < 0.01) but not at 100 µg/mL (55.00 ± 22.63%) (Figure 2e). 

Taken together, all fucoidans were protective against oxidative stress-induced reduction of viability, and all showed a similar pattern, with the highest viability rates at 10 and 50 µg/mL. However, the fucoidans displayed significant differences when their effects were compared. LD fucoidan clearly showed the strongest protective effect, which was significantly higher than that of SL (for 1 and 10 µg/mL *p* < 0.001; 50 µg/mL *p* < 0.001), significantly higher than that of FE (1 µg/mL *p* < 0.01; 10–100 µg/mL *p* < 0.001), and significantly higher than FS (all *p* < 0.001). FV was significantly more effective than FE (1 µg/mL *p* < 0.05; 10–100 µg/mL *p* < 0.01) and significantly more effective than FS (all *p* < 0.001). Finally, SL was significantly more protective than FE (1 µg/mL *p* < 0.05; 10 µg/mL *p* < 0.01; 50 µg/mL *p* < 0.001; 100 µg/mL *p* < 0.01) and more protective than FS (all *p* < 0.001). FE and FS, however, displayed no statistically significant differences (Table 1). Ranging the protective effect, LD > FV > SL > FE > FS. 

#### 2.1.2. ARPE19 Cells

ARPE19 cells are an immortal RPE cell line. One important role of RPE cells is protection against oxidative stress [18]. We tested the appropriate concentration of H_2_O_2_ for the following experiments, but ARPE19 cells turned out to be rather resistant to oxidative stress induced by hydrogen peroxide, which is consistent with the literature [19]. Treatment with H_2_O_2_ induced a significant reduction in cell viability, detected in ANOVA. While incubation with 100 and 200 µM H_2_O_2_ did not induce any significant reduction in cell viability compared to the control (100 µM 11.45 ± 9.65%; 200 µM 96.07 ± 14.75%), 400 and 1000 µM H_2_O_2_ significantly reduced cell survival (400 µM 86.75 ± 18.62%, *p* < 0.01; 1000 µM 76.2 ± 22.74%, *p* < 0.001). However, none of these concentrations reduced cell survival to approximately 50% (Figure 1b). We then tested tert-Butyl hydroperoxide (TBHP) on ARPE19 cells, which induced a significant reduction in cell viability (detected in ANOVA) and showed a concentration-dependent effect with a significant reduction of cell viability at 500 µM (100 µM 97.24 ± 11.56%, 250 µM ± 22.66%; 500 µM 55.33 ± 15.3%) (Figure 1c). Therefore, for the following experiments, a concentration of 500 µM TBHP was chosen.

**Figure 3:**
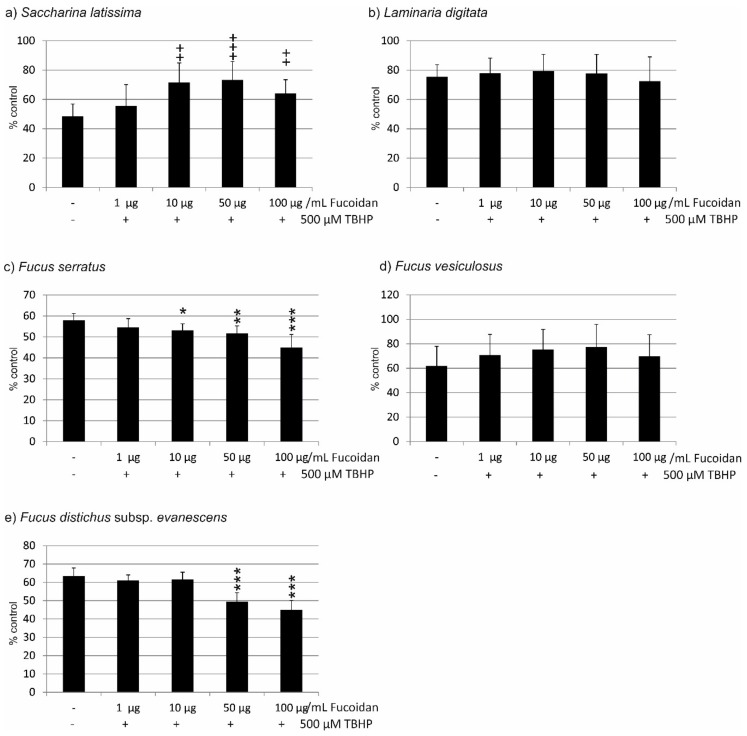
Cell viability of ARPE19 cells challenged with 500 µM TBHP after incubation with fucoidan from (**a**) *Saccharina latissima* (SL), (**b**) *Laminaria digitata* (LD), (**c**) *Fucus serratus* (FS), (**d**) *Fucus vesiculosus* (FV), (**e**) *Fucus distichus* subsp. *evanescens* (FE). Cell viability was measured by MTS assay and is depicted as mean and standard deviation, with the control set as 100%. Only SL fucoidan displayed a protective effect, while FS and FE fucoidans reduced cell viability. Significance was evaluated with Friedman’s ANOVA and subsequent Student’s *t*-test, ++ *p* < 0.01, +++ *p* < 0.001, for protective effects against 500 µM TBHP, * *p* < 0.05, ** *p* < 0.01 and *** *p* < 0.001 for exacerbating effects against 500 µM TBHP (*n* = 8).

In the experiments concerning the fucoidan of *Saccharina latissima*, incubation with 500 µg/mL TBHP induced a reduction in cell viability to 48.50 ± 8.44%. ANOVA testing showed significant differences between the groups. Incubation with 1 µg/mL induced no significant protection (55.63 ± 14.48%), while 10 µg/mL, 50 µg/mL, and 100 µg/mL significantly increased cell viability (71.50 ± 13.37%, *p* < 0.01; 73.25 ± 12.59%, *p* < 0.001; 64.00 ±9.46%, *p* < 0.01) (Figure 3a). In the experiments testing fucoidan from *Laminaria digitata*, incubation with 500 µg/mL TBHP induced a reduction in cell viability to 75.50 ± 8.20%. ANOVA testing showed statistically significant differences between the groups. However, none of the administered concentrations of fucoidan protected the cells against loss of cell viability (1 µg/mL 77.88 ± 10.40%; 10 µg/mL 79.38 ± 11.33%; 50 µg/mL 77.75 ± 12.94%; 100 µg/mL 72.50 ± 16.55%) (Figure 3b). Testing fucoidan from *Fucus serratus*, incubation with 500 µM TBHP resulted in a reduction of cell viability to 58.00% (±3.24). ANOVA testing showed statistically significant differences between the groups. Again, none of the tested concentrations of FS fucoidan conferred any protection. On the contrary, 10–100 µg/mL FS fucoidan significantly reduced the viability of the cells (1 µg/mL 54.50 ± 4.21%; 10 µg/mL 53.13 ± 3.18%, *p* < 0.05; 50 µg/mL 51.75 ± 3.56%, *p* < 0.01; 100 µg/mL 44.88 ± 6.27%. *p* < 0.001) (Figure 3c). When testing for fucoidan from *Fucus vesiculosus*, incubation with 500 mM TBHP resulted in a reduction of cell viability to 62.00% (±15.86). ANOVA testing showed statistically significant differences between groups. However, none of the tested concentrations of FV fucoidan induced a significant change in cell viability (1 µg/mL 70.88 ± 16.80%; 10 µg/mL 75.38 ± 16.43%; 50 µg/mL 77.50 ± 18.4%; 69.75 ± 17.69%) (Figure 3d). Finally, when testing fucoidan from *Fucus distichus* subsp. *evanescens*, incubation with 500 mM TBHP resulted in a reduction of cell viability to 63.50% (±4.33). ANOVA testing showed statistically significant differences between groups. While FE fucoidan in concentrations of 1 µg/mL (61.00 ± 3.08%) and 10 µg/mL (61.50 ± 4.06%) displayed no influence on cell viability, both 50 µg/mL and 100 µg/mL FE fucoidan significantly reduced cell viability (50 µg/mL 49.38 ± 4.99%; 100 µg/mL 45.00 ± 5.15%; both *p* < 0.001) (Figure 3e). 

Taken together, the results differ profoundly from those found with OMM-1 cells, with only *Saccharina latissima* showing protection, *Laminaria digitata* and *Fucus vesiculosus* displaying no influence, and *Fucus serratus* and *Fucus distichus* subsp. *evanescens* showing an additional toxic effect on the cells. 

In direct comparison, SL fucoidan is significantly different from LD fucoidan (1 µg/mL; *p* < 0.01), from FS fucoidan (10 µg/mL (*p* < 0.01)–100 µg/mL (*p* < 0.001), and from FE fucoidan (50 µg/mL and 100 µg/mL, both *p* < 0.001). The differences between SD fucoidan and FV fucoidan are not significant. Similar results are obtained with LD fucoidan, which does not significantly differ from FV fucoidan, but does differ from FE fucoidan and FS fucoidan in all concentrations tested (FE: 1–10 µg/mL *p* < 0.01, 50–100 µg/mL *p* < 0.001; FS 1–50 µg/mL *p* < 0.001, 100 µg/mL *p* < 0.01). FV fucoidan differs from FE fucoidan at 10–100 µg/mL (10 µg/mL *p* < 0.05; 50–100 µg/mL *p* < 0.01) and from FS fucoidan at all concentrations (1 µg/mL *p* < 0.05, 10–100 µg/mL *p* < 0.01). FE fucoidan differs from FS fucoidan in the lower concentrations (1 µg/mL *p* < 0.01, 10 µg/mL *p* < 0.001) (Table 2).

### 2.2. VEGF Secretion

The effect of the five different fucoidans on the secretion of VEGF was compared in two different RPE cell types, the human cell line ARPE19 and primary RPE cells derived from porcine eyes. Untreated ARPE19 cells secreted considerably and significantly less VEGF, with a mean of 416.33 pg/mL (±415.27), in a collection time of 24 h compared to primary porcine RPE cells, which secreted a mean of 2386.88 pg/mL (±824.81) (*p* < 0.001) in a collection time of 4 h, as tested in ANOVA and subsequent *t*-test. 

#### 2.2.1. ARPE19

**Figure 4:**
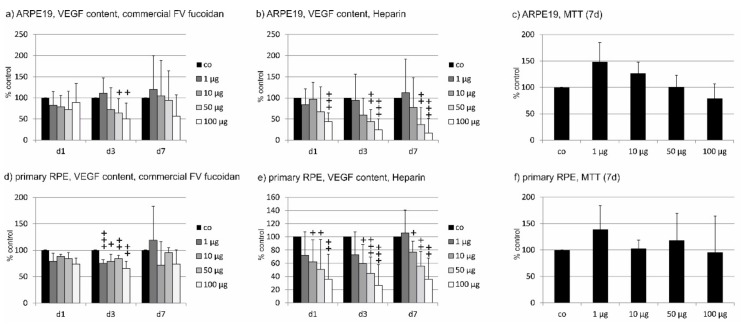
Effect of incubation time for vascular endothelial growth factor (VEGF) experiments. Commercially available fucoidan from *Fucus vesiculosus* was applied for 1 day (d1), 3 days (d3), and 7 days (d7), respectively, at indicated concentrations, to ARPE19 (**a**) or primary porcine retinal pigment epithelium (RPE) (**d**). In addition, heparin was tested on ARPE19 (**b**) and RPE cells (**e**). Cell viability was tested after 7 days (**c**,**f**). VEGF reduction was primarily seen after 3 days of incubation. Heparin showed a dose-dependent effect with similar significant reductions at all tested time points for concentrations of 10–100 µg/mL. No toxic effect was seen after 7 days in either cell type. Significance was evaluated with Friedman’s ANOVA and subsequent Student’s *t*-test, + *p* < 0.05 ++ *p* < 0.01, +++ *p* < 0.001 against the control (*n* = 4–6).

Before testing the five fucoidan extracts, we established the parameters for this test with commercially available fucoidan from *Fucus vesiculosus* (Sigma) with both ARPE19 cells and primary RPE cells (see below). Sigma fucoidan was applied to the cells and supernatant was harvested after 1 day, 3 days, and 7 days. After 7 days, the experiment was terminated and cell viability was measured. Using Sigma fucoidan on ARPE19 cells for one day, no statistical difference could be found in ANOVA (1 µg/mL 82.13 ± 33.16%; 10 µg/mL 79.13 ± 27.21%; 50 µg/mL 72.25 ± 43.1%; 100 µg/mL 89.13 ± 45.02%). After 3 days of incubation, a strong VEGF reduction could be seen, which was significant in ANOVA and was significant at concentrations of 50 µg/mL and 100 µg/mL in further testing (1 µg/mL 110.83 ± 35.94%; 10 µg/mL 71.83 ± 52.07%; 50 µg/mL 63.83 ± 34.62%, *p* < 0.05; 100 µg/mL 50.33 ± 37.33%, *p* < 0.05; day 7: 1 µg/mL 120 ± 79.39%; 10 µg/mL 105.00 ± 83.01%; 50 µg/mL 93.83 ± 69.61%; 100 µg/mL 56.83 ± 50.33%) (Figure 4 a). In addition, we tested the effect of heparin as a known VEGF-binding control compound. At all days, a significant reduction could be detected in ANOVA testing. At the first day, a significant reduction was found for a concentration of 100 µg/mL heparin (1 µg/mL 84.25 ± 37.56%; 10 µg/mL 96.50 ± 40.33%; 50 µg/mL 67.50 ± 58.41%; 100 µg/mL 44.13 ± 20.18%, *p* < 0.001). For day 3 and day 7, both 50 µg/mL and 100 µg/mL displayed a significant reduction of VEGF (day 3: 1 µg/mL 94.17 ± 61.81%; 10 g/ml 59.50 ± 40.01%; 50 µg/mL 43.50 ± 28.39%, *p* < 0.01; 100 µg/mL 24.50 ± 26.30%, *p* < 0.001; day 7: 1 µg/mL 112.33 ± 79.45%; 10 µg/mL 77.67 ± 70.13%; 50 µg/mL 36.83 ± 40.01%, *p* < 0.01; 100 µg/mL 17.17 ± 34.40%, *p* < 0.001) (Figure 4b). No significant reduction in cell viability could be detected after 7 days (1 µg/mL 148.17 ± 37.12%; 10 µg/mL 127.00 ± 20.98%; 50 µg/mL 101.00 ± 21.93%; 100 µg/mL 78.83 ± 28.11%) (Figure 4c).

Based on these results, we investigated VEGF content after 3 days of incubation with fucoidan for all following experiments. VEGF content was normalized to cell viability and displayed in relation to an untreated control.

**Figure 5:**
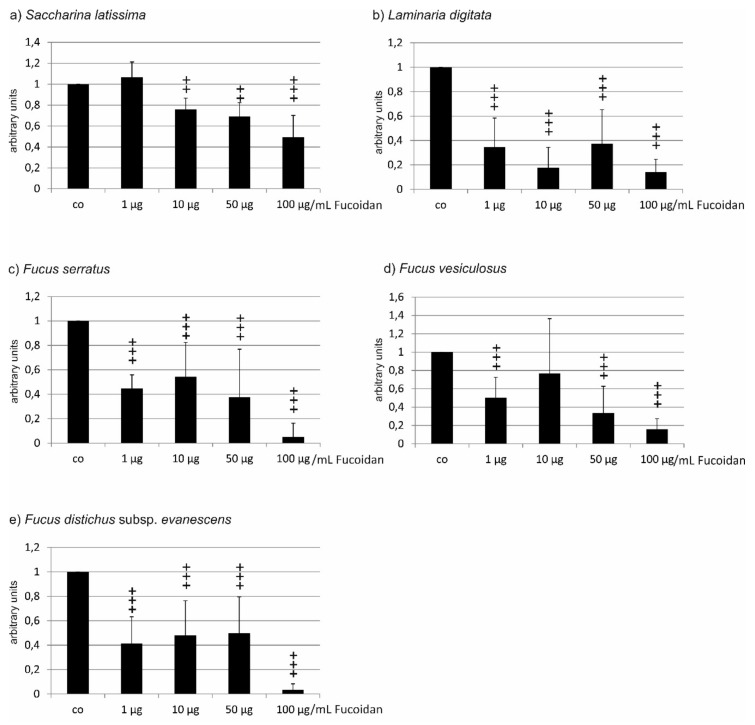
VEGF secretion in ARPE19 cells after incubation with different concentrations of fucoidan. (**a**) *Saccharina latissima* (SL), (**b**) *Laminaria digitata* (LD), **c**) *Fucus serratus* (FS), (**d**) *Fucus vesiculosus* (FV), (**e**) *Fucus distichus* subsp. *evanescens* (FE). VEGF content was evaluated by ELISA and normalized to cell viability. Control = 1. In ARPE19 cells, all fucoidans reduced VEGF content with the efficacy of LD > FS > FE > FV > SL. Significance was evaluated with Friedman’s ANOVA and subsequent Student’s *t*-test, ++ *p* < 0.01, +++ *p* < 0.001 compared to the control (*n* = 6–8).

After 3 days, fucoidan from all algae tested showed a significant reduction in ANOVA. Fucoidan from *Saccharina latissima* showed a reduction of VEGF when applied in concentrations of 10–100 µg/mL (1 µg/mL 1.07 ± 0.15; 10 µg/mL 0.76 ± 0.11, *p* < 0.01; 50 µg/mL 0.69 ± 0.13, *p* < 0.01; 100 µg/mL 0.46 ± 0.21, *p* < 0.001) (Figure 5a). Fucoidan from *Laminaria digitata* significantly reduced VEGF content in all concentrations tested (1 µg/mL 0.35 ± 0.24; 10 µg/mL 0.18 ± 0.17; 50 µg/mL 0.38 ± 0.28; 100 µg/mL 0.14 ± 0.11; all *p* < 0.001) (Figure 5b). Similarly, all concentrations of fucoidan from *Fucus serratus* showed significant inhibition of VEGF (1 µg/mL 0.45 ± 0.11; 10 µg/mL 0.54 ± 0.28; 50 µg/mL 0.38 ± 0.39; 100 µg/mL 0.05 ± 0.11; all *p* < 0.001) (Figure 5c). Fucoidan from *Fucus vesiculosus* reduced VEGF at concentrations 1 µg/mL (0.50 ± 0.22, *p* < 0.001) and 50–100 µg/mL (50 µg/mL 0.33 ± 0.29; 100 µg/mL 0.16 ± 0.15, both *p* < 0.001), but not 10 µg/mL (0.77 ± 0.60) (Figure 5d). Fucoidan from *Fucus distichus* subsp. *evanescens* reduced VEGF at all concentrations tested (1 µg/mL 0.41 ± 0.22; 10 µg/mL 0.48 ± 0.28; 50 µg/mL 0.50 ± 0.30; 100 µg/mL 0.04 ± 0.05; all *p* < 0.001) (Figure 5e). 

In ARPE19 cells, all tested fucoidans reduced the amount of secreted VEGF, with the highest concentration (100 µg/mL) generally displaying the strongest effect. The differences between the different fucoidans are not as profound as those found in protective effects and are generally limited to effects seen at certain concentrations. LD fucoidan differs only slightly from FV or FS fucoidan (both 10 µg/mL, *p* < 0.05) or from FE fucoidan (10 µg/mL and 100 µg/mL both *p* < 0.05), while there were significant differences compared to SL fucoidan in all concentrations tested (1–10 µg/mL *p* < 0.001; 50 µg/mL *p* < 0.05; 100 µg/mL *p* < 0.01). SL fucoidan significantly differed from FV fucoidan in all concentrations but 10 µg/mL (1 µg/mL *p* < 0.001; 50 µg/mL *p* < 0.05; 100 µg/mL *p* < 0.01), while it differed from FE and FS fucoidan at concentrations of 1 µg/mL and 100 µg/mL (both *p* < 0.001). FV fucoidan did not display any significant differences from FS fucoidan, and only at 100 µg/mL when compared to FE fucoidan (*p* < 0.05). Finally, FE and FS fucoidan did not display any significant differences (Table 3). Ranging the effect on VEGF secretion results in LD > FS > FE > FV > SL.

#### 2.2.2. Primary RPE

The pretesting of Sigma fucoidan on cells for 1 day, 3 days, and 7 days led to the following. In primary porcine RPE cells, we found no significant reduction of VEGF content in the supernatant after 1 day (1 µg/mL 79.25 ± 15.04%; 10 µg/mL 88.00 ± 4.69%; 50 µg/mL 84.25 ± 11.87%; 100 µg/mL 74.25 ± 11.18%). After 3 days, however, a significant reduction could be detected in ANOVA and was found in all concentrations assessed (1 µg/mL 75.50 ± 6.46%, *p* < 0.001; 10 µg/mL 78.75 ± 13.33%, *p* < 0.05; 50 µg/mL 84.00 ± 6.83%; *p* < 0.01; 100 µg/mL 65.5 ± 13.92%, *p* < 0.01). This effect was no longer seen after 7 days (1 µg/mL 119.00 ± 64.18%; 10 µg/mL 71.75 ± 44.70%; 50 µg/mL 95.75 ± 9.07%); 100 µg/mL 73.75 ± 27.21%) (Figure 4d). In addition, we have tested the effect of heparin as a known VEGF binding control compound. At all three tested days, a significant reduction could be detected in ANOVA and concentrations of 10, 50, and 100 µg/mL heparin significantly reduced VEGF in a dose-dependent manner (day 1: 1 µg/mL 72.167 ± 35.32%; 10 µg/mL 62.17 ± 33.44%, *p* < 0.05; 50 µg/mL 50.83 ± 44.91%, *p* < 0.05: 100 µg/mL 36.00 ± 37.70%, *p* < 0.01; day 3: 1 µg/mL 73.17 ± 34.11%; 10 µg/mL 59.60 ± 28.78%, *p* < 0.05; 50 µg/mL 44.50 ± 25.11%, *p* < 0.001; 100 µg/m. 26.33 ± 31.40%, *p* < 0.001; day 7: 1 µg/mL 106.00 ± 34.58%; 10 µg/mL 76.60 ± 16.88%, *p* < 0.05; 50 µg/mL 55.80 ± 22.00%, *p* < 0.01; 100 µg/mL 36.00 ± 31.98%, *p* < 0.01) (Figure 4e). No significant reduction of cell viability could be seen after 7 days (1 µg/mL 139.17 ± 45.20%; 10 µg/mL 103.16 ± 15.38%; 118.00 ± 51.54%; 100 µg/mL 95.50 ± 68.63%) (Figure 4f). Based on these results, we investigated VEGF content after 3 days for all following experiments. VEGF content was normalized to cell viability and displayed in relation to untreated controls. 

**Figure 6:**
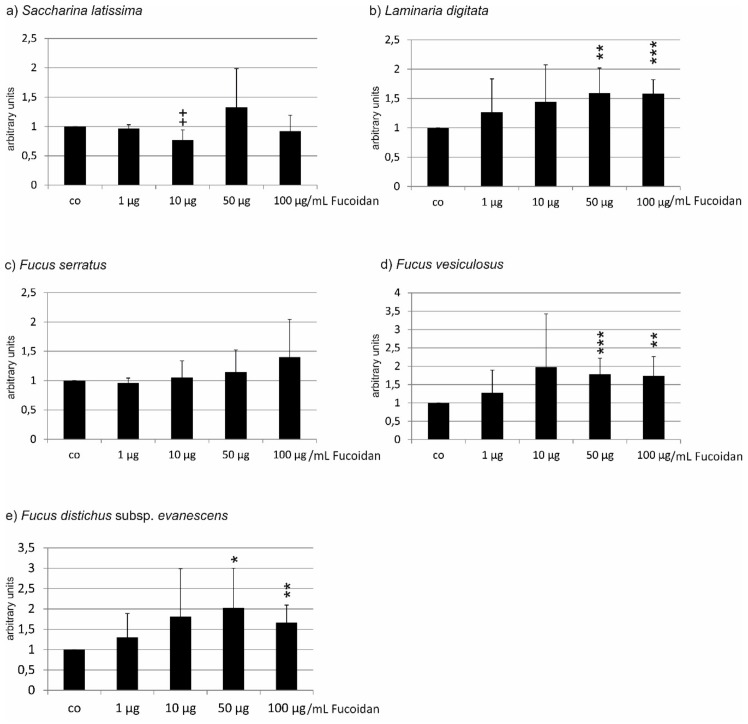
VEGF secretion of primary porcine RPE cells after incubation with different concentrations of fucoidan from (**a**) *Saccharina latissima* (SL), (**b**) *Laminaria digitata* (LD), (**c**) *Fucus serratus* (FS), (**d**) *Fucus vesiculosus* (FV), (**e**) *Fucus distichus* subsp. *evanescens* (FE). VEGF content was evaluated in ELISA and normalized to cell viability. Control = 1. In RPE cells, only SL fucoidan reduced the VEGF content of RPE cells (10 µg/mL), while LD, FV, and FE induced a higher signal at concentrations of 50 and 100 µg/mL. Significance was evaluated with Friedman’s ANOVA and subsequent Student’s *t*-test, + *p* < 0.05 reduction compared to the control, * *p* < 0.05, ** *p* < 0.01 and *** *p* < 0.001 (*n* = 7).

After 3 days, fucoidan from *Saccharina latissima* reduced the VEGF content of RPE cells at a concentration of 10 µg/mL (0.77 ± 0.17, *p* < 0.01), while the other concentrations showed no significant effect (1 µg/mL 0.97 ± 0.07; 50 µg/mL 1.33 ± 0.66; 100 µg/mL 0.92 ± 0.27) (Figure 6a). None of the other fucoidans reduced VEGF content in RPE cells. Fucoidan from *Laminaria digitata* did not reduce VEGF secretion in primary porcine RPE cells in any concentration tested; however, a significant increase was detected in ANOVA, significantly increasing the VEGF signal at concentrations of 50 µg/mL and 100 µg/mL (1 µg/mL 1.27 ± 0.57; 10 µg/mL 1.44 ± 0.63; 50 µg/mL 1.59 ± 0.43, *p* < 0.01; 1.58 ± 0.24, *p* < 0.001) (Figure 5b). Fucoidan from *Fucus serratus* displayed no significant influence on VEGF secretion in primary porcine RPE at any concentration tested (1 µg/mL 0.96 ± 0.08; 10 µg/mL 1.05 ± 0.28; 50 µg/mL 1.14 ± 0.38; 100 µg/mL 1.40 ± 0.64) (Figure 6c). Fucoidan from *Fucus vesiculosus* did not reduce VEGF in any tested concentration but, similar to LD fucoidan, displayed a significant increase in ANOVA testing, increasing the VEGF signal at concentrations of 50 and 100 µg/mL (1 µg/mL 1.28 ± 0.62; 10 µg/mL 1.97 ± 1.46; 50 µg/mL 1.78 ± 0.44, *p* < 0.001; 100 µg/mL 1.74 ± 0.52, *p* < 0.01) (Figure 6d). Fucoidan from *Fucus distichus* subsp. *evanescens* displayed similar results. While no reduction of VEGF content could be seen at any tested concentration, significant differences could be detected in ANOVA, with 50 µg/mL and 100 µg/mL displaying an increase in VEGF content (1 µg/mL 1.31 ± 0.59; 10 µg/mL 1.81 ± 1.18; 50 µg/mL 2.02 ± 0.98, *p* < 0.05; 100 µg/mL 1.67 ± 0.43, *p* < 0.01) (Figure 6e). 

In primary RPE cells, only *Saccharina latissima* showed a reductive effect on VEGF content in the supernatant. When comparing the different fucoidans, significant differences were only found between SL and LD fucoidan (at a concentration of 10 µg/mL) and between FV and FS fucoidan (at a concentration of 50 µg/mL) (Table 4). 

### 2.3. Binding Affinity to VEGF

In the pathogenesis of the exudative form of AMD, VEGF is an important factor [5,6]. VEGF inhibitors are currently used as a therapy standard [11]. As has been known for a long time that heparin and other sulfated polysaccharides have a high affinity for VEGF [20], fucoidans may not only reduce the secretion of VEGF but also directly antagonize its actions. Therefore, we investigated the binding affinity of fucoidans to VEGF in comparison to heparin, and aimed to identify those fucoidans with the strongest binding affinity to VEGF.

**Figure 7:**
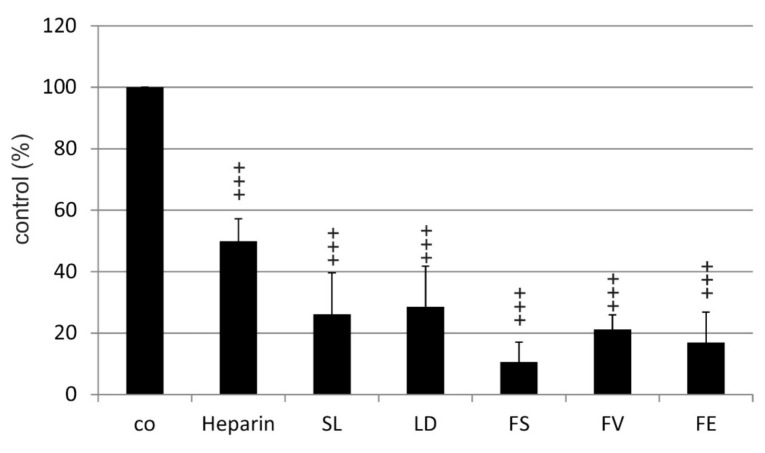
VEGF binding affinity of fucoidans from *Saccharina latissima* (SL), *Laminaria digitata* (LD), *Fucus serratus* (FS), *Fucus vesiculosus* (FV), *Fucus distichus* subsp. *evanescens* (FE), and of heparin. Significance was evaluated with Friedman’s ANOVA and subsequent Student’s *t*-test, +++ *p* < 0.001 compared to control (biotinylated heparin).

In the competitive VEGF binding assay used, the replacement of biotinylated heparin by the test compound was measured. At a concentration of 0.5 µg/mL, maximum binding of biotinylated heparin was achieved (data not shown). By adding increasing concentrations of unlabeled heparin, the detection of biotinylated heparin decreased (data not shown). As expected, incubation with a mixture of 0.5 µg/mL heparin and 0.5 µg/mL biotinylated heparin reduced the signal to 49.96% (±7.28) and thus the binding of biotinylated heparin to VEGF. All five fucoidans showed a significant affinity to VEGF compared to the control, tested in ANOVA and subsequent testing (SL 26.13 ± 13.46%; LD 28.57 ± 13.20%; FS 10.63 ± 6.43%; FV 21.27 ± 4.69% and FE 16.97 ± 9.91%) (Figure 7). Moreover, the three *Fucus* fucoidans bound significantly more strongly to VEGF than heparin (*p* < 0.01) (Table 5). Although FS and FE showed a slightly higher affinity, there were no significant differences between the five fucoidans (Table 5).

## 3. Discussion

Fucoidans are of great interest to biomedical research, especially considering their possible application in age-related macular degeneration [4]. However, the effects of fucoidans can profoundly vary depending on their origin and method of extraction. Therefore, generalized statements about their activities should be avoided. 

In this study, we have compared fucoidans from five different brown algae species harvested in summer and obtained from the same supplier (Coastal Research & Management, Kiel, Germany). The preparation of the algal material as well as the extraction and purification of the fucoidans were performed in parallel according to a standardized procedure. Therefore, this study is well equipped to reliably examine the effects of fucoidans from different algal species. The aim was to compare the beneficial properties of fucoidans with regard to age-related macular degeneration.

In our study, we focused on two major factors important for the development of age-related macular degeneration, i.e., oxidative stress and VEGF. While interference with these is without doubt of great interest, we are aware that other activities, e.g., anticomplementary, anti-inflammatory effects, or influence on lipid metabolism, are also potentially beneficial for impairing AMD pathogenesis and are of high interest for further testing.

We have previously shown that OMM-1 cells are protected by commercially available fucoidan [17]. In contrast to uveal melanoma cells, RPE cells are intrinsically strongly protected against oxidative stress [18], so we tested oxidative stress protection both in OMM-1 cell lines, which we know could be protected by fucoidans, having demonstrated this for commercial fucoidan from *Fucus vesiculosus* [17], as well as the RPE cell line ARPE19. All five fucoidans protected OMM-1 cells from oxidative stress, confirming our previous results. However, fucoidans can differ in their properties depending on the test system or the cell types due to distinct cellular and molecular pathways. A major task of RPE cells is oxidative stress protection [18] and, as mentioned above, they are naturally highly resistant to oxidative stress. ARPE19 cells, as an RPE cell line, consequently behaved differently from OMM-1 cells, not only concerning their susceptibility to oxidative stress itself, but also their reaction to fucoidans. In contrast to OMM-1 cells, only SL fucoidan displayed a protection of cell viability, while FE and FS fucoidan even exacerbated the effect.

Little is known about oxidative stress pathways in uveal melanoma cells, but an increased susceptibility due to the reduction of superoxide dismutase activity in uveal melanoma cells has been described [21]. There is a debate about the direct reactive oxygen species (ROS) scavenging effect of fucoidans. The ROS scavenging potency of fucoidan has been described in several publications [22,23,24,25], but it has recently been demonstrated in cell-free systems that these measured effects are mainly due to co-extracted phenolic and terpenoid compounds in the fucoidan preparations [15,26]. Generally, the scavenging effect of fucoidan against hydrogen peroxide has been described to be rather weak [24,25]. Our results even suggest that the in vitro ROS scavenging activity may be irrelevant as, in contrast to SL and LD, the three *Fucus* fucoidans showed an ROS scavenging effect (manuscript in preparation) but did not protect ARPE19 cells from oxidative stress. 

However, independent of all of the results from simple cell-free assays, fucoidans may exhibit antioxidative activity by cellular effects. Accordingly, fucoidan has been shown to increase the expression of superoxide dismutase (SOD) in several experimental models and activate the transcription factor nuclear factor erythroid 2-like 2 (Nrf2), the “master regulator” of the antioxidative stress response [27,28,29,30,31,32]. Both the overall protective effect of our fucoidans on OMM-1 and the limited protective effect on ARPE19 cells (only found for *Saccharina latissima* fucoidan) could be explained via these pathways. As mentioned above, uveal melanoma cells have been described to have a reduced SOD activity [21]. Hence, the SOD-inducing effect of fucoidans could protect these cells against an oxidative stress insult. In contrast, RPE cells have a high intrinsic stress response level mediated by Nrf2 [18]. Indeed, knock-out of Nrf2 renders RPE cells highly susceptible to oxidative stress insults [33] and Nrf2 knock-out mice develop AMD-like features at an older age [34]. It is feasible that these protective pathways are already at maximum efficacy, so that any further enhancement by fucoidans may be impossible. Therefore, the different effects of fucoidans on the two cell lines are assumed to be due to distinct impacts on the respective cellular pathways. Further research needs to be conducted to elucidate these pathways. It should be noted, however, that oxidative stress protection in AMD is not only needed for (the rather resistant) RPE cells, but also for the rather fragile photoreceptor cells [35]. Therefore, the effect of fucoidans on oxidative stress-induced photoreceptor cell death should also be evaluated in further studies. 

A major contributor to the pathology of wet AMD is the growth factor VEGF, and its inhibition is the only current treatment option for AMD patients. We have previously shown that commercially obtained fucoidan from *Fucus vesiculosus* additively reduced VEGF expression when co-applied with the VEGF inhibitor bevacizumab [3]. On the molecular level, the interaction of fucoidan with VEGF differs profoundly from that of the current therapeutic anti-VEGF molecules. VEGF antibody-derived compounds bevacizumab and ranibizumab, as well as the fusion protein aflibecept, interact with specific amino acids in the receptor-binding domain of VEGF, causing a steric inhibition of the binding of VEGF to its receptor [36], with differences in affinities between the compounds [37]. The interaction of fucoidan and other heparin-related compounds is complex, however, depending on features such as sulfation and molecular weight [38,39]. Furthermore, fucoidan has been shown to also have a binding affinity to VEGF receptors and to facilitate the internalization of VEGF receptors, blocking the binding and in-vitro functions of VEGF [38,40,41].

In our current study, we were able to demonstrate antagonization as well as a reduction of VEGF secretion in ARPE19 cells, in which all five fucoidans were effective. In primary porcine RPE cells, however, only SL displayed a significant effect. 

Fucoidan was found to influence Stat3-regulated promoters, which includes the promoter of VEGF [42]. But it should be noted that we have previously shown that in unchallenged primary RPE cells, Stat3 is not involved in constitutive VEGF expression [43]. Therefore, this mechanism of fucoidan-mediated VEGF reduction is not feasible. We have also previously shown that VEGF is positively regulated in an autocrine way via the VEGFR-2 [43,44], whereby fucoidan has been shown to bind to VEGF165 and to competitively inhibit the interaction of VEGF with VEGFR2 [39,40]. This pathway has been suggested to be involved in VEGF reduction mediated by fucoidan [3,40]. Such an extracellular mode of action for fucoidans is now supported by the binding of the five fucoidans to VEGF. Their affinity was significantly higher than that of heparin, whereas heparan sulfate was not able to reduce the binding of biotinylated heparin to VEGF. Interaction of VEGF with heparin sulfate on the cell surface was found to be involved in effective VEGFR2 activation [45]. Thus, the fucoidans may competitively prevent this interaction and thus attenuate signaling through VEGFR2, resulting in reduced VEGF expression and secretion. In line with this assumed mode of action are the findings that the intraocular injection of heparan sulfate or heparin in mice eyes with aberrant angiogenesis results in reduced neovascularization [46]. Given the even higher affinity to VEGF of fucoidans, this seems promising.

The amount of VEGF secreted by primary RPE cells in this study was much higher than that of ARPE19 cells (596.72 pg/h for primary RPE vs. 17.35 pg/h for ARPE19; factor 34.4). In the presence of such high VEGF concentrations, the VEGF antagonizing mode of action obviously became ineffective, explaining the discrepant results in ARPE19 cells and RPE cells. Therefore, the effect of SL on VEGF secretion in primary RPE cells is even more remarkable.

Our data clearly show a positive effect of the tested fucoidans in terms of oxidative stress protection and VEGF inhibition, with the most promising fucoidan extracted from *Saccharina latissima*. Among the tested fucoidans, SL had the highest degree of sulfation, the highest molecular weight, and the highest degree of purity (under submission). But these parameters cannot explain its superiority or the ranking of the other fucoidans. Fucoidans from *Saccharina latissima* have been previously shown to be highly biologically active compared to those from other brown algae species [15,16]. Furthermore, in line with our finding concerning VEGF inhibition, fucoidans from *Saccharina latissima* have been shown to inhibit angiogenesis in tumor models [47]. However, it seems too early to decide that the other fucoidans are not worth further investigation. Other activities beneficial for AMD therapy should be regarded as well. Further preclinical and clinical research is warranted, but fucoidans may be a potential treatment option for age-related macular degeneration. To develop potential therapeutics from fucoidan, in addition to finding the most suitable source and a sustainable and reliable harvest and extraction method, bioavailability and application forms need to be tested. 

In addition, VEGF secretion and oxidative stress are also involved in the pathomechanisms of diabetic retinopathy [48,49]. Therefore, fucoidans may also be of great interest for diabetic patients, especially considering that fucoidan may also reduce blood glucose levels and ameliorate hypertension [4]. 

In conclusion, we compared fucoidan from five brown algae species in terms of three activities that are considered promising for the treatment of AMD, i.e., their capacity for oxidative stress protection, inhibition of VEGF secretion, and binding affinity to VEGF. Based on these three basic parameters, the fucoidan from *Saccharina latissima* turned out to be most suitable for further investigations. 

## 4. Material and Methods

### 4.1. Cell Culture

The uveal melanoma cell line OMM-1 [50] was a kind gift from Dr. Sarah Coupland and was cultivated in an appropriate medium (RPMI, Merck, Darmstadt, Germany, supplemented with 10% fetal calf serum and 1% penicillin/streptomycin). The immortal human RPE cell line ARPE19 was obtained from American Type Culture Collection (ATCC) and cultivated in an appropriate medium (Dulbecco’s Modified Eagle’s Medium (DMEM), supplemented with penicillin/streptomycin (1%), non-essential amino acids (1%), 4-(2-hydroxyethyl)-1-piperazineethanesulfonic acid (HEPES) (25%), and 10% fetal calf serum).

Primary RPE cells were prepared as previously described [51,52]. In brief, RPE cells were harvested from cleaned porcine eyes by trypsin incubation and cultivated in an appropriate medium (DMEM, HyClone, Thermo Fisher Sc., Bremen, Germany, supplemented with penicillin/streptomysin (1%), HEPES (25%), non-essential amino acids (1%), all Merck, Darmstadt, Germany, and 10% fetal calf serum, Linaris GmbH, Wertheim-Bettingen, Germany). RPE and ARPE19 cells were used at confluence and OMM-1 at 80% confluence for further experimentation.

### 4.2. Fucoidans

Fucoidans were extracted from dried stocks of the species *Saccharina latissima* (SL; Atlantic, Funningsfjord, Faroe Island), *Fucus vesiculosus* (FV; Baltic, Kiel Bay, Germany), *Laminaria digitata* (LD; Atlantic; Churchbay, Island ), *Fucus distichus* subsp. *evanescens* (FE; Baltic, Kiel Cana, Germany), and *Fucus serratus* (FS; Baltic, Kiel Bay, Germany), all harvested in summer and provided by Coastal Research & Management, Kiel, Germany. The fucoidans were extracted as previously described [53]. Briefly, the pulverized algal material was defatted by Soxhlet extraction with 99% (*v*/*v*) ethanol, and was then extracted with aqueous 2% calcium chloride for 2 h at 85 °C under reflux conditions. The supernatants of the raw extracts were concentrated and precipitated with ice-cold ethanol in a final concentration of 60% (*w*/*w*). After centrifugation, the sediments were dissolved in demineralized water, dialyzed, and lyophilized. In addition, commercially available fucoidan from Sigma (Sigma-Aldrich, Deisenhofen, Germany, F8190) was used. Fucoidan was solved in Ampuwa bidest (Fresenius, Schweinfurt, Germany), and then further diluted with appropriate cell medium for the cell experiments and phosphate buffered saline (PBS) for the VEGF binding assay, filtered through a 0.2 µm filter (Sarstedt, Nümbrecht, Germany), and applied to the cells in final concentrations of 1, 10, 50, and 100 µg/mL. 

### 4.3. Oxidative Stress

#### 4.3.1. OMM-1

OMM-1 cells were treated with hydrogen peroxide (H_2_O_2_) to induce oxidative stress-related cell death, as previously shown [17]. As OMM-1 is a cancer cell line that may change its characteristics during subculture, we evaluated the appropriate concentrations of H_2_O_2_ resulting in approximately 50% cell viability. In order to assess this, OMM-1 cells were treated with different concentrations (100, 200, 400, 1000 mM) of H_2_O_2_ for 24 h and cell viability was investigated by MTS assay (see below). To investigate the potential protective effects of the different fucoidans, a concentration of 1 mM H_2_O_2_ was chosen. Cells were treated with fucoidan (1, 10, 50, and 100 µg/mL) 30 min prior to the application of H_2_O_2_. 

#### 4.3.2. ARPE19

Corresponding experiments to find the appropriate H_2_O_2_ concentration for ARPE19 cells revealed that none of the tested concentrations of H_2_O_2_ (100 µM, 200 µM, 400 µM, 1000 µM) induced a cell death of about 50% after 24 h, as detected by MTS assay. In addition, the cell death rate was highly variable after H_2_O_2_ incubation (see results). Therefore, we tested tert-Butyl hydroperoxide (TBHP), a more stable inducer of oxidative stress in RPE cells [33], at concentrations of 100 µM, 250 µM, and 500 µM, for 24 h and investigated cell viability by MTS assay, as described below. In order to investigate the potential protective effect of the different fucoidans, a concentration of 500 µM TBHP was chosen. Cells were treated with fucoidan 30 min prior to the insult.

### 4.4. Methyl Thiazolyl Tetrazolium (MTT) Assay

MTT assay is a common method in cell research [54] and was conducted as previously described [3]. In brief, after treatment with the fucoidans, the cells were washed and incubated with 0.5 mg/mL MTT (dissolved in DMEM without phenol red). After removal and further washing of the cells, cells were lysed with dimethyl sulfoxide (DMSO) and the absorbance was measured at 550 nm with a spectrometer (Elx800, BioTek, Bad Friedrichshall, Germany). 

### 4.5. MTS Assay

The MTS assay is a commercially available viability assay and was used according to the manufacturers’ instructions (CellTiter 96^®^ AQueous One Solution Cell Proliferation Assay (Promega, Mannheim, Germany)). The cell viability assay was performed in 96 well plates in phenol red-free medium with the same supplements described above. In each well, 20 µL of MTS solution was added for 1 h.

### 4.6. VEGF ELISA

VEGF was detected in the supernatants of ARPE19 and primary RPE cells using commercially available ELISA kits (R&D Systems, Wiesbaden, Germany) according to the manufacturer’s instructions. To establish the parameters of VEGF ELISA, we investigated time-dependent VEGF secretion in ARPE19 and primary RPE cells in the presence and absence of commercially available fucoidan from *Fucus vesiculosus* (Sigma-Aldrich, F8190) for 1 day, 3 days, and 7 days. A cell viability assay (MTT) was conducted after 7 days. According to these results, an incubation time of 3 days was chosen for experiments with the five different fucoidans. The medium was changed 24 h prior in ARPE19 cells and 4 h prior in primary RPE cells and the supernatant collected. Measured VEGF content was normalized for cell survival and is depicted in relation to that of untreated control cells.

### 4.7. Competitive VEGF Binding Assay 

The affinity to VEGF of the test compounds was investigated with a competitive VEGF-binding assay using biotinylated heparin. In addition to the fucoidans, heparin (EDQM, no. Y0001282, Strasbourg, France) was tested.

The wells of a 96-well Nunc-Immuno MaxiSorp microplate (Sigma-Aldrich, Deisenhofen, Germany) were coated with 0.1 µg recombinant human VEGF 165 (R&D Systems Cat. 293-VE/CF) dissolved in 100 µL PBS overnight at 4 °C. After washing with PBS, the coated wells were blocked for 90 min at 37 °C with 100 µL of 5 mg/mL bovine serum albumin (BSA, dissolved in PBS) and subsequently washed three times with PBS. During the blocking, 65 µL of 1 µg/mL heparin, biotin conjugate (Merck, Darmstadt, Germany) in PBS, and 65 µL of 1 µg/mL test compounds in PBS were preincubated at 4 °C. For the blank and the 100% binding value, 100 µL PBS and 100 µL of 0.5 µg/mL biotinylated heparin in PBS, respectively, were preincubated at 4 °C. Aliquots of 100 µL of these solutions were pipetted into the coated microplate wells and incubated for 2 h at 37 °C with gentle agitation. After three washing steps, 100 µL of streptavidin alkaline phosphate conjugate (Southern Biotech/, Birmingham, AL, USA, stock solution diluted 1:3000 with PBS) was incubated for 1 h at 37 °C with gentle agitation. The next steps involved three washings with PBS and incubation with 100 µL p-nitrophenyl phosphate substrate system (Sigma-Aldrich, Deisenhofen, Germany) for 30 min in the dark. The reaction was stopped by addition of 25 µL 3 N NaOH, and the absorbance was measured at 405 nm. Blank values in the absence of biotinylated heparin were subtracted from the measured values. The reduction of the binding of biotinylated heparin by the test compounds is indicated as a percentage in relation to the binding of the biotinylated heparin alone. 

### 4.8. Statistics

All experiments testing fucoidans were independently repeated at least six times, experiments for establishing oxidative stress response were repeated at least three times, and the VEGF binding experiments were performed in duplicates on three different days. Statistics were calculated using Statistica 7 (Statsoft, Tulsa, OK, USA) and Microsoft Excel (Excel 2010, Microsoft, Redmond, WA, USA). A Friedman’s ANOVA was performed, and, if a significant difference between groups was detected, a subsequent Student’s *t*-test was conducted. A *p* value of <0.05 was considered significant. All bars represent mean and standard deviation.

## Figures and Tables

**Table 1:** Comparison of the protective effects of the different fucoidans at different concentrations against oxidative stress cell death in OMM-1 cells induced with 1 mM H_2_O_2_.

Compared Fucoidans	1 µg/mL	10 µg/mL	50 µg/mL	100 µg/mL
**LD vs. FV**	not significant (ns)	ns	ns	ns
**LD vs. SL**	*p* < 0.001	*p* < 0.001	*p* < 0.01	ns
**LD vs. FE**	*p* < 0.05	*p* < 0.001	*p* < 0.001	*p* < 0.001
**LD vs. FS**	*p* < 0.001	*p* < 0.001	*p* < 0.001	*p* < 0.001
**FV vs. SL**	ns	ns	ns	ns
**FV vs. FE**	*p* < 0.05	*p* < 0.01	*p* < 0.01	*p* < 0.01
**FV vs. FS**	*p* < 0.001	*p* < 0.001	*p* < 0.001	*p* < 0.001
**SL vs. FE**	*p* < 0.05	*p* < 0.01	*p* < 0.001	*p* < 0.01
**SL vs. FS**	*p* < 0.001	*p* < 0.001	*p* < 0.001	*p* < 0.001
**FE vs. FS**	ns	ns	ns	ns

**Table 2:** Comparison of the protective effects of the different fucoidans at different concentrations after oxidative stress cell death in ARPE19 cells induced by 500 µM TBHP.

Compared Fucoidans	1 µg/mL	10 µg/mL	50 µg/mL	100 µg/mL
**LD vs. FV**	ns	ns	ns	ns
**LD vs. SL**	*p* < 0.01	ns	ns	ns
**LD vs. FE**	*p* < 0.01	*p* < 0.01	*p* < 0.001	*p* < 0.001
**LD vs. FS**	*p* < 0.001	*p* < 0.001	*p* < 0.001	*p* < 0.01
**FV vs. SL**	ns	ns	ns	ns
**FV vs. FE**	ns	*p* < 0.05	*p* < 0.01	*p* < 0.01
**FV vs. FS**	*p* < 0.05	*p* < 0.01	*p* < 0.01	*p* < 0.01
**SL vs. FE**	ns	ns	*p* < 0.001	*p* < 0.001
**SL vs. FS**	ns	*p* < 0.01	*p* < 0.001	*p* < 0.001
**FE vs. FS**	*p* < 0.01	*p* < 0.001	ns	ns

**Table 3:** Comparison of the effect of the different fucoidans at different concentrations on VEGF secretion in ARPE19 cells.

Compared Fucoidans	1 µg/mL	10 µg/mL	50 µg/mL	100 µg/mL
**LD vs. FV**	ns	*p* < 0.05	ns	ns
**LD vs. SL**	*p* < 0.001	*p* < 0.001	*p* < 0.05	*p* < 0.01
**LD vs. FE**	ns	*p* < 0.05	ns	*p* < 0.05
**LD vs. FS**	ns	*p* < 0.05	ns	ns
**FV vs. SL**	*p* < 0.001	ns	*p* < 0.05	*p* < 0.01
**FV vs. FE**	ns	ns	ns	*p* < 0.05
**FV vs. FS**	ns	ns	ns	ns
**SL vs. FE**	*p* < 0.001	ns	ns	*p* < 0.001
**SL vs. FS**	*p* < 0.001	ns	ns	*p* < 0.001
**FE vs. FS**	ns	ns	ns	ns

**Table 4:** Comparison of the effect of different fucoidans at different concentrations on VEGF secretion in primary porcine RPE cells.

Compared Fucoidans	1 µg/mL	10 µg/mL	50 µg/mL	100 µg/mL
**LD vs. FV**	ns	ns	ns	ns
**LD vs. SL**	ns	*p* < 0.05	ns	ns
**LD vs. FE**	ns	ns	ns	ns
**LD vs. FS**	ns	ns	ns	ns
**FV vs. SL**	ns	ns	ns	ns
**FV vs. FE**	ns	ns	ns	ns
**FV vs. FS**	ns	ns	*p* < 0.05	ns
**SL vs. FE**	ns	ns	ns	ns
**SL vs. FS**	ns	ns	ns	ns
**FE vs. FS**	ns	ns	ns	ns

**Table 5:** Comparison of the VEGF binding affinity of fucoidans and heparin.

Compared Substances	Significance
Heparin vs. FV	*p* < 0.01
Heparin vs. FS	*p* < 0.01
Heparin vs. FE	*p* < 0.01
Heparin vs. LD	ns
Heparin vs. SL	ns
LD vs. FV	ns
LD vs. SL	ns
LD vs. FE	ns
LD vs. FS	ns
FV vs. SL	ns
FV vs. FE	ns
FV vs. FS	ns
SL vs. FE	ns
SL vs. FS	ns
FE vs. FS	ns
